# Acute inflammatory demyelinating polyneuropathy or Guillain-Barré syndrome associated with COVID-19: a case report

**DOI:** 10.1186/s13256-021-02831-4

**Published:** 2021-04-28

**Authors:** Dmitriy A. Gagarkin, Keith E. Dombrowski, Keyur B. Thakar, John C. DePetrillo

**Affiliations:** 1 Phelps Family Medicine Residency Program, 701 North Broadway, 755 Building/Suite 405, Elmsford, Sleepy Hollow, Tarrytown, NY 10591 USA; 2 University of South Florida/Tampa General Hospital, Tampa, USA; 3 Hematology/Oncology, Phelps Hospital Northwell Health, Tarrytown, NY USA; 4 Intensive Care Unit, Phelps Hospital Northwell Health, Tarrytown, NY USA

**Keywords:** COVID-19, Coronavirus infections, Guillain-Barré syndrome, Case report

## Abstract

**Background:**

Coronavirus disease 2019 (COVID-19) is a global pandemic. The disease, typically characterized by bilateral pulmonary infiltrates and profound elevation of inflammatory markers, can range in severity from mild or asymptomatic illness to a lethal cytokine storm and respiratory failure. A number of recognized complications of COVID-19 infection are described in the literature. Common neurological complications include headache and anosmia. Guillain-Barré syndrome (GBS) is an uncommon complication described in isolated case reports. However, a causal relationship has yet to be established. This case report adds to the growing body of evidence that GBS is a potential COVID-19 complication.

**Case presentation:**

A 70-year-old Caucasian woman with recently diagnosed COVID-19 infection presented to the emergency department with 4 days of gradually worsening ascending lower extremity weakness. Exam revealed bilateral lower extremity weakness, mute reflexes, and sensory loss. Soon after starting intravenous administration of immunoglobulin (IVIG), the patient developed respiratory distress, eventually requiring intubation. She remained intubated for the duration of her IVIG treatment. After five rounds of treatment, the patient was successfully extubated and transferred to acute rehab. Following 4 weeks of intense physical therapy, she was able to walk with assistance on room air.

**Conclusion:**

At the present time, this is one of the few reports of acute inflammatory demyelinating polyneuropathy (AIDP) or GBS associated with COVID-19 in the United States. It is unclear whether a causal relationship exists given the nature of the syndrome. However, in light of the growing number of reported cases, physicians should be aware of this possible complication when evaluating COVID-19 patients.

## Background

Coronavirus disease 2019 (COVID-19) is a global pandemic. The disease, typically characterized by bilateral pulmonary infiltrates and profound elevation of inflammatory markers, can range in severity from mild or asymptomatic illness to lethal cytokine storm and respiratory failure [1]. A number of recognized complications of COVID-19 infection are described in the literature, including thromboembolic [2–4], cardiovascular [5, 6], and neurological events [7]. Common neurological complications include headache and anosmia. Guillain-Barré syndrome (GBS) is an uncommon complication described in isolated case reports [8–10]. However, a causal relationship has yet to be established. This case report adds to the growing body of evidence supporting Guillain-Barré as a potential COVID-19 complication.

## Case presentation

A 70-year-old Caucasian woman presented to the emergency department (ED) with 4 days of gradually worsening ascending lower extremity weakness. Three weeks prior to presentation, she was seen at the same ED for complaints of fever and cough. At that time she was diagnosed with COVID-19 via a nasal polymerase chain reaction (PCR) specimen and advised to maintain self-isolation. Her respiratory symptoms resolved within a week, at which point she developed bilateral calf pain with associated paresthesias. Over the course of a few days the distribution of the paresthesias progressed from her feet to her trunk, at which point she was no longer able to stand. This weakness prompted her repeat visit to the hospital. Her medical history was significant for stage I breast cancer, gastroesophageal reflux disease (GERD), osteoporosis, and hypothyroidism. Her home medications included anastrozole, benzonatate, famotidine, levothyroxine, and apixaban for prophylactic anticoagulation in a COVID-19 patient. She denied ever smoking or drinking alcohol. She was retired, lived with her husband, and denied any known sick contacts or international travel.

On presentation, the patient was hypertensive (177/100 mmHg), afebrile (97.8 °F), and breathing comfortably (18 breaths per minute; oxygen saturation 98% on room air), with normal heart rate (78 beats per minute). Weight upon admission was 65 kg. Physical examination revealed an elderly woman, no toxicity, in no acute distress, without tachypnea or use of accessory muscles. Pulmonary auscultation revealed clear lungs bilaterally. Neurological examination revealed bilateral lower extremity weakness (strength 3/5), mute reflexes, stocking-glove sensory loss to pinprick and cold up to above the knees and elbows, and decreased vibratory sensation in the fingers and medial malleolus with intact proprioception.

Initial laboratory results demonstrated a neutrophil-predominant leukocytosis with elevated inflammatory markers.

**Table:** Serum laboratory findings with institution-specific reference range

White blood cell (WBC) count	13,700 white blood cells per mm^3^ (normal range 4500–11,000 WBC/mm^3^)
D-dimer	349 ng/mL (normal range < 229 ng/mL)
C-reactive protein	22.7 mg/L (normal range ≤ 4 mg/L)
Lactate dehydrogenase	879 U/L (normal range 50–242 U/L)
Ferritin	395 ng/mL (normal range 15–150 ng/mL)

**Fig. 1:**
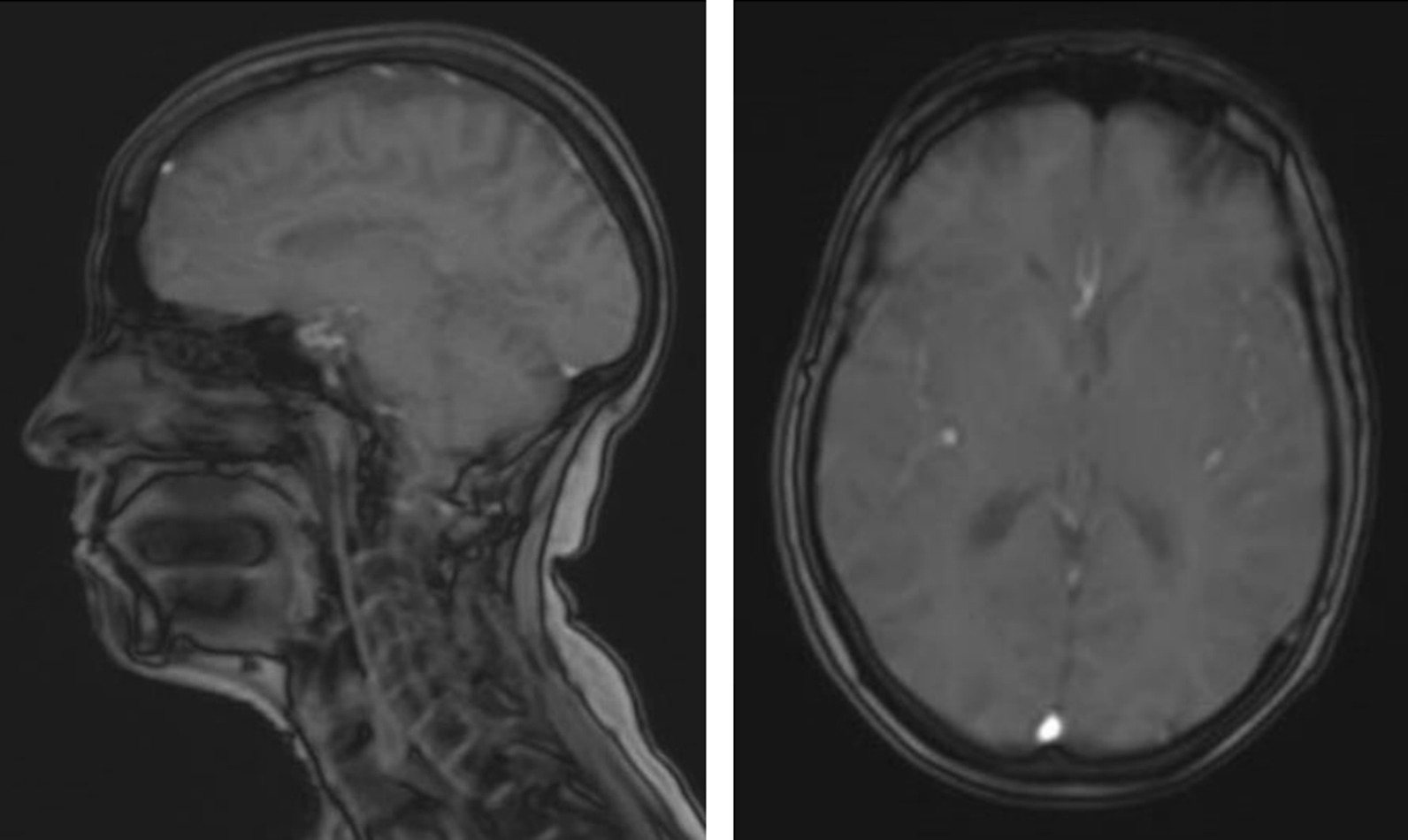
Magnetic resonance imaging findings

Chest X-ray and head computed tomography were unremarkable. Brain magnetic resonance imaging revealed only mild small vessel ischemic disease of the white matter, with no significant focal lesions. PCR of her nasopharyngeal swab was positive for severe acute respiratory syndrome coronavirus 2 (SARS-CoV-2) (Fig. 1).

Based on the clinical and laboratory findings, a preliminary diagnosis of GBS was made and the patient was started on daily intravenous immunoglobulin (IVIG) administration at a dose of 0.4 g/kg on the day of admission. Lumbar puncture was not performed due to the heavy burden of the pandemic and lack of resources at the time. Diagnosis of Guillain-Barré, however, was clinically supported by the rapid progression of symptoms, mute deep tendon reflexes, and bilateral presentation. Soon after her first dose of IVIG, the patient developed dysphagia with respiratory distress and hypoxia requiring oxygen supplementation (presumably due to the worsening of her underlying GBS and intolerance of oral secretions, given no evidence of volume overload or allergic response). She was intubated and transferred to the intensive care unit (ICU). Based on institutional protocols at the time, she was also treated with hydroxychloroquine (800 mg oral loading dose followed by 400 mg daily for a total of 5 days), doxycycline (100 mg every 12 hours by mouth for 10 days), and enoxaparin (40 mg daily subcutaneous injection).

The patient remained intubated for the duration of her IVIG treatment, with one unsuccessful extubation. She completed five IVIG treatments and was transferred to an acute rehabilitation facility after successful ventilator liberation. At that time the patient's dysphagia had improved, but sensory and motor deficits persisted and were likely exacerbated by deconditioning from the ICU stay. Following 4 weeks of intense physical therapy, the patient was able to walk with assistance without supplemental oxygen. Neurological exam revealed improvement in strength to 4/5 in all extremities. Dysphagia improved. She was eventually discharged home with a home health aid, oral anticoagulation (given emerging findings of hypercoagulability in COVID-19 patients), and outpatient follow-up.

## Discussion

Guillain-Barré Syndrome refers to a collection of immune-mediated polyneuropathies, so named after the authors of the original description of the syndrome. Although the original understanding of GBS implied a single disorder, modern understanding of GBS recognizes several variant forms. The major forms of GBS are acute inflammatory demyelinating polyradiculopathy (AIDP), Miller Fisher syndrome (MFS), acute sensorimotor axonal neuropathy (AMSAN), and acute motor axonal neuropathy (AMAN).

While the exact cause of GBS remains unknown, one proposed mechanism is an antecedent infection that stimulates the immune response and leads to production of cross-reactive antibodies against the nervous system, a phenomenon known as molecular mimicry [11]. These cross-reactive antibodies can target the myelin or axons of the peripheral nerves, resulting in neurological deficits characteristic of the syndrome. Immune reactions against axonal membranes present as AMSAN or AMAN, while targeting myelin via cross-reactivity with Schwann cell epitopes can cause AIDP, as seen in our patient.

The cross-reactivity hypothesis is supported by various studies, many of which have focused on elucidating the mechanism of GBS in association with *Campylobacter jejuni* infection. Interestingly, there appear to be shared ganglioside antigens between peripheral nerves and the lipo-oligosaccharide coat of *Campylobacter jejuni* [12]. Furthermore, infections with *Campylobacter* can generate antibodies against specific gangliosides of the nervous system [13]. Although speculation at this point, it is possible and may even be likely that a pathogen with structures just similar enough to those of the host can stimulate an immune response against both simultaneously.

AIDP classically presents as progressive symmetric muscle weakness (usually lower extremity) with absent or diminished deep tendon reflexes and paresthesia of the hands and feet. Facial nerve palsies and oropharyngeal weakness are also present in about half of the patients [11, 14]. Although less common, weakness of respiratory muscles requiring intubation and ventilatory support can be seen in 10–30% of AIDP patients [15]. We suspect that our patient developed both oropharyngeal and respiratory muscle weakness which eventually required intubation to maintain adequate oxygenation. Given the normal chest x-ray and lack of concerning clinical findings, alternative explanations such as COVID-19 pneumonia, fluid overload, or allergic/anaphylactic reaction are unlikely.

While the initial diagnosis of GBS can be based on cardinal clinical presentation, lumbar puncture and electrodiagnostic studies are often performed on patients with suspected GBS to help confirm the diagnosis. Classical findings in GBS include a lumbar puncture with elevated cerebrospinal fluid (CSF) protein and a normal white blood cell count (known as albuminocytologic dissociation) as well as abnormal patterns on nerve conduction studies (NCS) and needle electromyography (EMG). While this case report lacks the CSF or nerve conduction studies to help support the diagnosis, the delayed onset of symptoms, prototypical progressive sensory and motor symptoms with areflexia, and response to immunotherapy is consistent with an ascending variant of AIDP.

Coronaviridae such as SARS-CoV-1 and Middle East respiratory syndrome coronavirus (MERS-CoV) have been associated with various neurological sequelae including encephalitis and seizures. Infrequently, these viruses have been associated with inflammatory polyneuropathy [16–18]. GBS and Bickerstaff brainstem encephalitis were both found to have occurred after MERS as apparent post-infectious conditions [18]. As in our patient, the neurological symptoms occurred days or weeks after the SARS-CoV-2 infection. As with other cases of AIDP, the origin of polyneuropathy is presumably a post-infectious immunological response to COVID-19.

## Conclusion

At this time, this is one of only a few reports of AIDP or GBS associated with COVID-19 in the United States. It is unclear whether a causal relationship exists given the nature of the syndrome. However, in light of the growing number of reported cases, physicians should be aware of this possible complication when evaluating COVID-19 patients. Further research is required to establish incidence and risk factors for the development of this complication.

## Data Availability

Not applicable.

## Abbreviations

AIDP: Acute inflammatory demyelinating polyneuropathy
AMAN: Acute motor axonal neuropathy
AMSAN: Acute sensorimotor axonal neuropathy
COVID-19: Coronavirus disease 2019
CSF: Cerebrospinal fluid
EMG: Electromyography
GBS: Guillain-Barré syndrome
GERD: Gastroesophageal reflux disease
ICU: Intensive care unit
IVIG: Intravenous immunoglobulin
MERS: Middle East respiratory syndrome
MFS: Miller Fisher syndrome
NCS: Nerve conduction studies
